# Blood Harmonisation of Endoscopic Transsphenoidal Surgical Video Frames on Phantom Models

**DOI:** 10.1109/ISBI56570.2024.10635809

**Published:** 2024-05-27

**Authors:** Mahrukh Saeed, Julien Quarez, Hassna Irzan, Bava Kesavan, Matthew Elliot, Oscar Maccormac, James Knight, Sebastien Ourselin, Jonathan Shapey, Alejandro Granados

**Affiliations:** ⋆ School of Biomedical Engineering and Imaging Sciences, King’s College London, UK; † Neurosurgery, King’s College Hospital, London, UK

**Keywords:** blood simulation, surgical videos, image harmonisation, physical phantom models, surgical training

## Abstract

Physical phantom models have been integral to surgical training, yet they lack realism and are unable to replicate the presence of blood resulting from surgical actions. Existing domain transfer methods aim to enhance realism, but none facilitate blood simulation. This study investigates the overlay of blood on images acquired during endoscopic transsphenoidal pituitary surgery on phantom models. The process involves employing manual techniques using the GIMP image manipulation application and automated methods using pythons Blend Modes module. We then approach this as an image harmonisation task to assess its practicality and feasibility. Our evaluation uses Structural Similarity Index Measure and Laplacian metrics. The results we obtained emphasize the significance of image harmonisation, offering substantial insights within the surgical field. Our work is a step towards investigating data-driven models that can simulate blood for increased realism during surgical training on phantom models.

## 1 Introduction

Endoscopic transsphenoidal surgery (ETSS) resects pituitary adenomas by inserting a surgical endoscope and instruments through the nose to reach the base of the skull [1]. Compared to other surgical interventions, ETSS requires a steep learning curve to acquire adequate proficiency for surgeons to be able to minimise risks and adverse events that could lead to intra- and post-operative complications such as cerebrospinal fluid leakage [2].

Physical phantom models are typically used by trainees to improve their dexterity and technical skills [3]. In the context of ETSS, phantom models are specifically designed to mimic the critical anatomical structures [4]. Two main limitations of phantom models are their lack of realistic visual appearances and their inability to reproduce hemorrhage during surgical training.

Data-driven methods have been proposed to increase the realistic appearance of surgical interventions on phantom models. *Hyperrealism*, for instance, generates realistic visual representations of phantom models in real-time by facilitating domain transfer between a real and a synthetic video dataset [3]. This approach is based on a deep learning model for domain transfer called CycleGAN [5]. While this method convincingly depicts a more realistic appearance of soft tissue and surgical tool interaction, it does not mimic the appearance and dynamics of blood. This limits the practical application of the method as a whole.

Other approaches have utilised machine learning models such as general adversarial networks and convolutional neural networks to capture the structure and semantics of composite images by overlaying contents from a foreground image onto a background image [6, 7]. Image harmonisation has also been proposed to augment the realism of an image overlaid onto another image by matching their visual appearances [8]. For instance, SinGAN takes as input a background image during training to generate sub-images at different scales that are robust enough to handle added noise [9]. Then, during the harmonisation process, SinGAN seamlessly merges a foreground input image with the background image used during training to generate a harmonised image that increases the realism of the foreground with the background across the different sub-scales.

The aim of this paper is to investigate the realism of blood overlaid on image frames extracted from ETSS videos recorded on phantom models via image harmonisation. This approach has the potential to enhance manual dexterity, hone surgical abilities, and considerably lower the risk of tissue damage during endoscopic procedures. To the best of our knowledge, we are the first to investigate how to authentically replicate the blood composition and texture appearance on endoscopic image frames.

## 2 Experimental Design

### 2.1 Dataset

A neurosurgeon performed ETSS at the Surgical and Interventional Engineering mock operating room at St. Thomas’ Hospital, London, UK, utilising the TNSBox by UpSurgeOn (https://www.upsurgeon.com/). This allowed us to have an endoscopic video throughout the entire procedure. We obtained internal ethical approval at King’s College London (HR/DP-22/23-34122). We retrospectively extracted images from the captured video to integrate blood into these frames. The image frames were resized to 348 *×* 280 pixels. Intraoperative tissue images and blood splatter images were obtained from the internet.

### 2.2 Manual and Automatic Blood Simulation on Endoscopic Image Frames

We designed a pipeline to allow us to manually identify and, later on, automatise the image processing steps required for blood simulation on endoscopic image frames. We used GIMP (https://www.gimp.org/) to manually blend the endoscopic image frame (background image), the intra-operative tissue image (foreground image), and blood splatters (overlaid images). Three images were manually generated, including 1) an endoscopic image frame with intra-operative tissue only, 2) an endoscopic image frame with blood splatters only, and 3) an endoscopic image frame with both intra-operative tissue and blood splatters. Subsequently, the manual image processing steps were automated via *Blend Modes* in Python. In this study, we employed the ‘multiplying’ blending mode to combine and overlay the blood splatter onto the endoscopic image frame to achieve the same results as with the manual pipeline.

### 2.3 Image Harmonisation of Blood Splatters on Endoscopic Image Frames

**Fig. 1:**
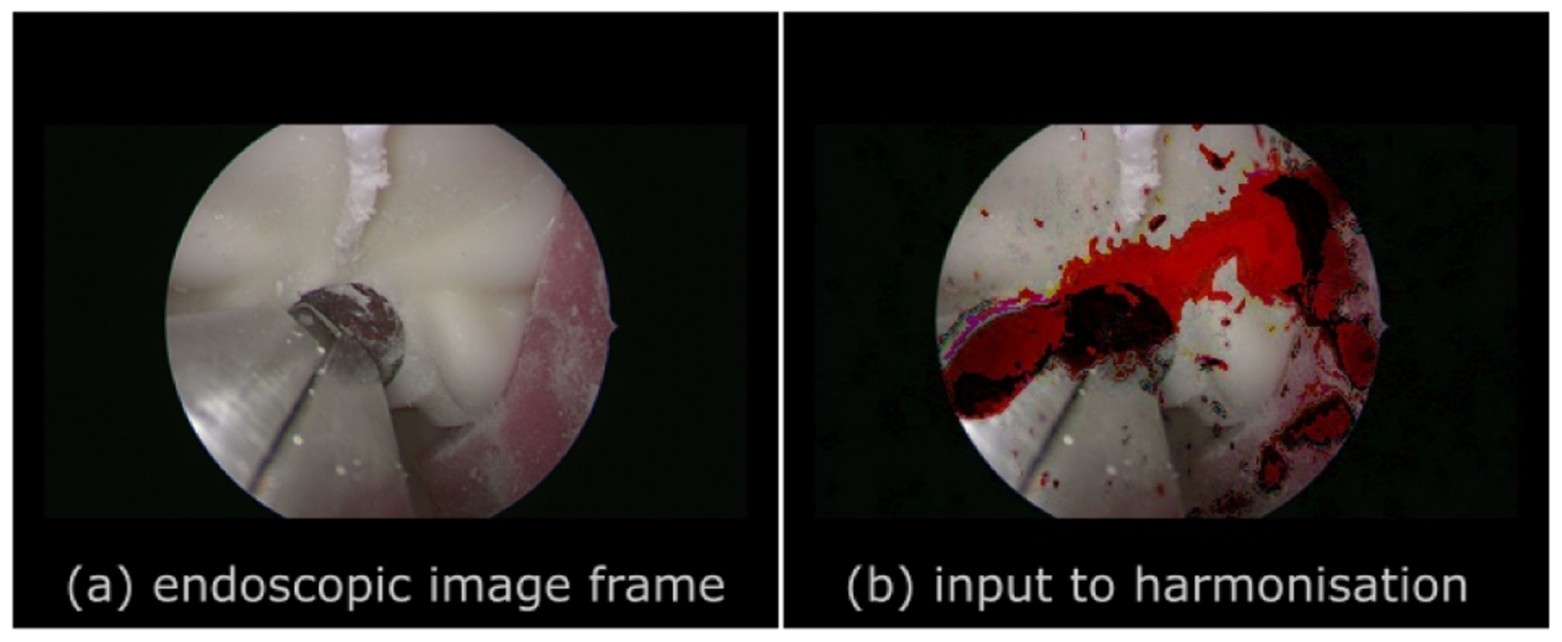
Two inputs are utilised to generate our harmonised results. Figure (a) serves as an input training image for the SinGAN model, whereas figure (b) serves as the input foreground image for the harmonisation task.

We employ SinGAN [9] to generate endoscopic image frames that incorporate blood splatters through image harmonisation. This allows us to investigate the effectiveness of blending these splatters with endoscopic image frames captured from phantom models. SinGAN is initially trained using an endoscopic image frame as input, illustrated in Fig.1(a), followed up by a harmonisation process with up to eight scales using six different blood splatters, one of which is shown in Fig.1(b). We trained the models on an NVIDIA A100 GPU card.

### 2.4 Evaluation

The algorithms’ harmonisation performance was quantitatively evaluated using three metrics. We computed the Structural Similarity Index Measure (SSIM) and the Multi-Scale Structural Similarity Index Measure (MS-SSIM) loss metrics to assess the structural and visual similarities between the harmonised input and output images, as well as the disparities present between them [10, 11]. To further evaluate the blending process, the Laplacian filter was utilised to measure image blurriness and sharpness [12]. These metrics were computed across the six different blood splatters. We report their mean and standard deviation.

## 3 Results and Discussion

### 3.1 Manual

**Fig. 2:**
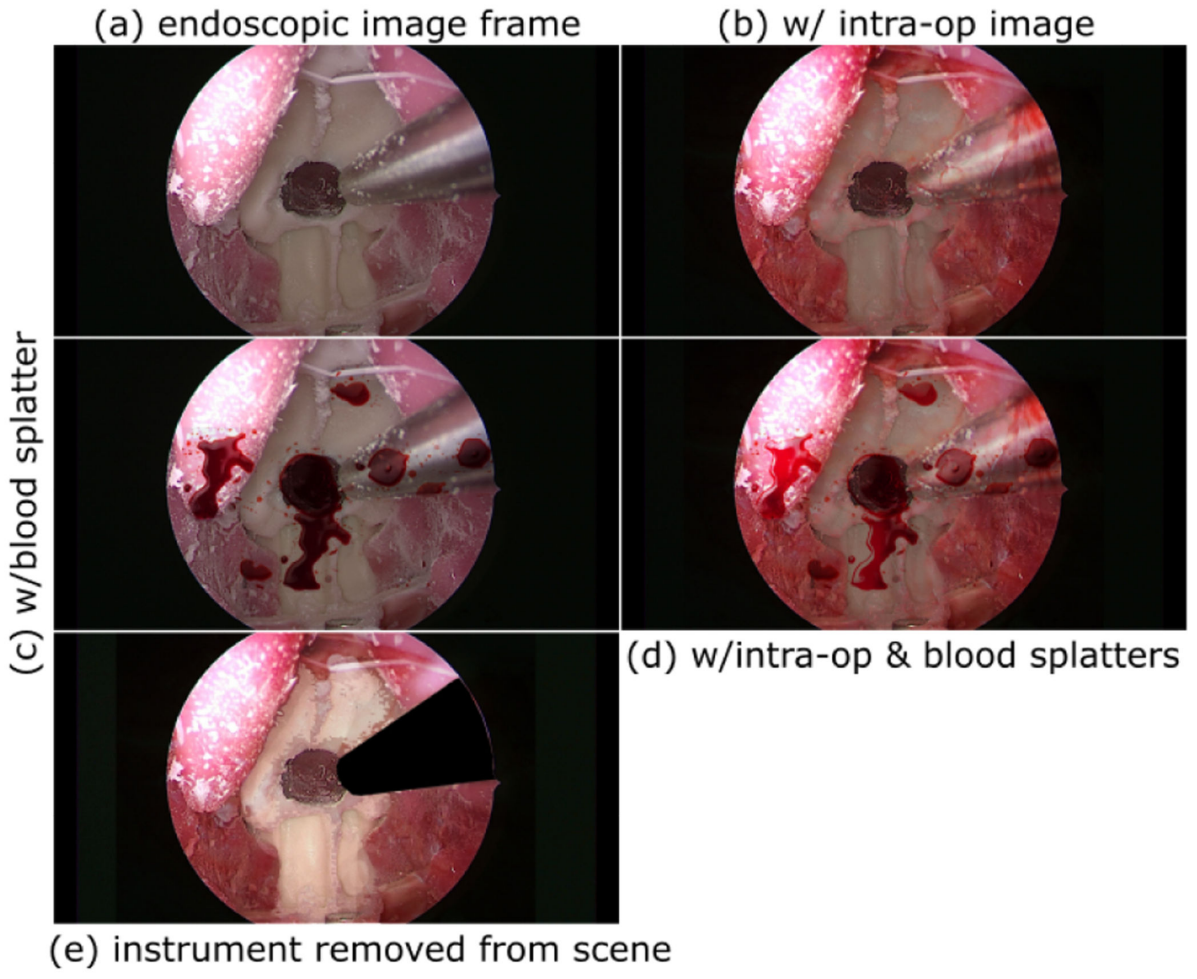
Manual overlay of blood. Starting with an endoscopic image frame (a), we use the GIMP application to assess the appearance and characteristics of a blood-simulated endoscopic image frame by: (b) overlaying intraoperative tissue, (c) overlaying blood splatters, and (d) overlaying both intraoperative tissue and blood splatters. (e) Results of automated image processing after applying *Blend Modes* to an image whereby the instrument has been first removed from the scene.

The image processing pipeline for increasing the realism and appearance of blood on endoscopic image frames is described below. We utilised the *fuzzy select* tool, modified the layer to *overlay* the intraoperative tissue layer, *multiply* the blood splatter layer, and set the overall opacity to 80%. The *dodge tool* was used to lighten specific areas of the blood splatters. These modifications provide an impression of the ‘look and feel’ of a blood-incorporated endoscopic image frame. The results produced by manual image processing are depicted in Fig. 2(b)-(d), illustrating the three modifications made to the endoscopic image frame.

### 3.2 Automatic

The automated *Blend Modes* module resulted in an image that was identical to Fig. 2(b), which served as our pre-processed image for this task. However, this image lacked realism due to the artificial blood depiction on non-tissue areas. In pursuit of heightened authenticity, the refinement process involved masking the instrument and then applying the blood simulation, as demonstrated in Fig. 2(e).

### 3.3 Image Harmonisation

**Fig. 3:**
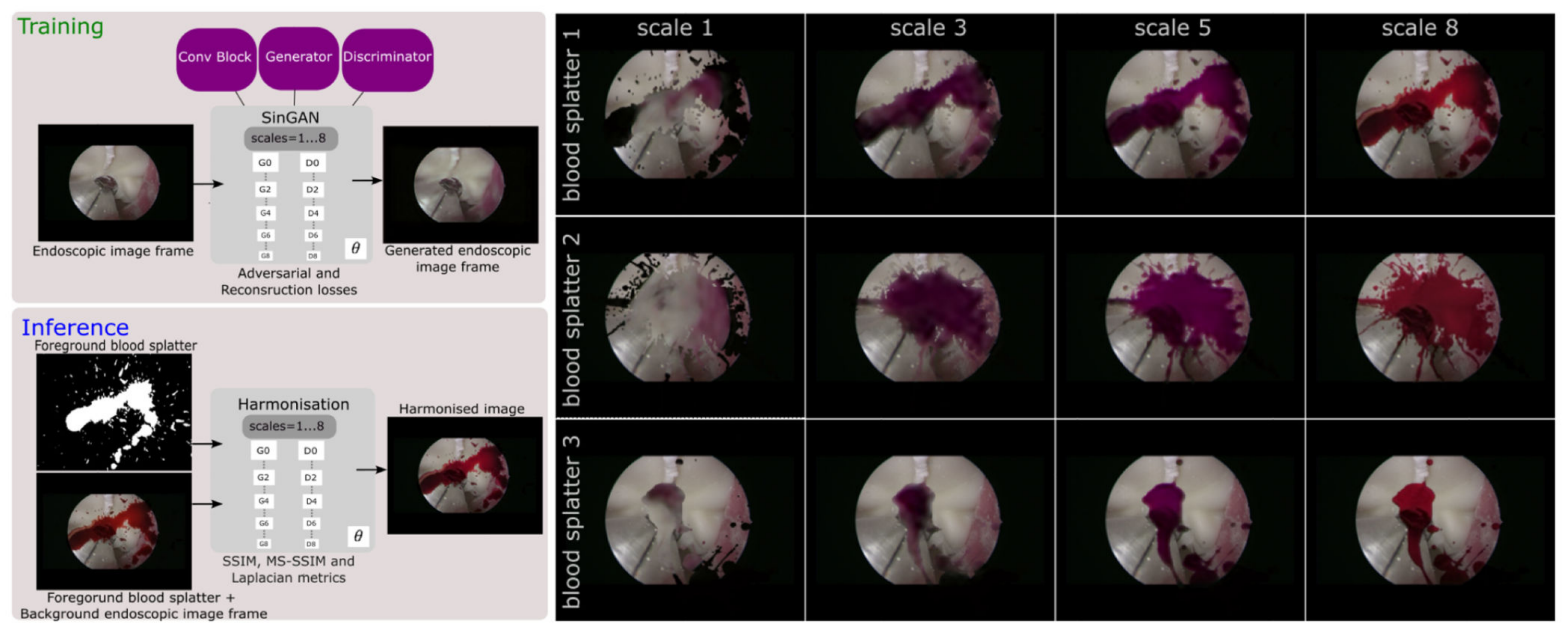
Qualitative comparison of three blood splatters after image harmonisation. The evaluation includes blood splatters 1, 2, and 3 each associated with scales 1,3, 5, and 8, representing the results acquired throughout the harmonisation process.

Upon training SinGAN, the harmonisation procedure was applied across eight different scales to showcase its progression as shown in Fig. 3 (left). As the scales increase, the endoscopic image frame progressively harmonises with the blood splatters. This effect is evident in Fig. 3 (right), highlighting the gradual enhancement of image cohesiveness as the scale increases. As explained by [10, 11], the high values observed for the SSIM and MS-SSIM statistics in Tab. 1a indicate that there were subtle structural and contrast similarities between the input and out images. In Tab. 1b, we provide the statistics for Laplacian metrics computed across the harmonised input and output images, including the harmonised output image’s foreground and background. The mean and standard deviation values of the output were evidently lower than those of the input. As identified in the study by [12], this indicates that the harmonisation task resulted in a smoother output image with finer textures, a reduction in intensity variations, and enhanced edges. Simultaneously, the background image exhibited higher mean and standard deviation values, demonstrating the successful preservation of many details of the ground truth image while blending the blood splatter foreground with the endoscopic background.

Although the harmonisation process might have improved the blending of blood splatters with the endoscopic image frame, our work has some limitations. First, the striking color contrast in the background and foreground images led to slight distortions and blurriness. This can be corrected by incorporating a temporal loss function across a specified window of image frames. Second, SinGAN needs to be trained fully from scratch for every single input image making this approach unfeasible for a real-time task. A generative model that could learn the dynamics [13] of blood from motion would likely address this limitation when working with videos. Last, blood splatters are not generated to account for the possible amounts and positions of blood subject to the interaction of tools with tissue. We foresee that the harmonisation of blood behaviour will be done in tandem with other approaches that automatically recognise surgical actions in real-time [14].

While our work is preliminary, in future work we consider the use of a latent diffusion model that generates overlaid images given the endoscopic image frame and dynamically generated blood masks as preconditioning to the generative model. Moreover, we aim to devise evaluation metrics specifically designed to assess the quality of harmonised images since there is currently no existing method for this purpose. Further studies are also required to investigate the effect of increased realism through blood simulation on the efficacy of surgical training and on the potential desensitisation to blood.

## 4 Conclusion

The primary objective of this initial study was to assess the realism of endoscopic image frames when incorporating the presence of blood manually, automatically, and via a recent model for image harmonisation. We investigate the feasibility of overlaying various blood splatter images on endoscopic image frames captured during ETSS. The significance of our work is a step towards enhancing surgical training on physical phantom models by integrating the dynamics of blood that result from tool-tissue interactions.

## Figures and Tables

**Table 1:** Evaluation metrics (SSIM, MS-SSIM, and Laplacian) and their respective mean and standard deviation for assessing the harmonised results across the six blood splatters.

	**Mean**	**Standard deviation**
SSIM	0.9456	0.005364
MS-SSIM	0.9901	0.002135
(a) SSIM and MS-SSIM		

	**Mean**	**Standard deviation**
Harmonised Input	460.9	156.3
Harmonised Output	288.3	76.49
Harmonised Foreground	1538	652.8
Harmonised Background	2187	883.6
(b) Laplacian		
